# Prognostic relevance and molecular correlates of Claudin-1 expression in pancreatic neuroendocrine tumors

**DOI:** 10.1016/j.humpath.2026.106208

**Published:** 2026-06-30

**Authors:** Antonin Fattori, Xiaofan Lu, Philippe Baltzinger, Philippe Bachellier, Pietro Addeo, Alessio Imperiale, Patrick Pessaux, Jean-Emmanuel Kurtz, Bernard Goichot, Thomas F. Baumert, Marie-Pierrette Chenard, Gabriel G. Malouf

**Affiliations:** a Department of Cancer and Functional Genomics, Institute of Genetics and Molecular and Cellular Biology, CNRS/ INSERM/UNISTRA, Illkirch, France; b Department of Pathology, Strasbourg University Hospitals, Strasbourg, France; c Department of Endocrinology, Diabetology and Nutrition, Strasbourg University Hospitals, Strasbourg, France; d Department of Hepato-Pancreato-Biliary Surgery and Liver Transplantation, Strasbourg University Hospitals, Strasbourg, France; e Department of Nuclear Medicine, Strasbourg University Hospitals, Strasbourg, France; f Department of Digestive and Endocrine Surgery, Strasbourg University Hospitals, Strasbourg, France; g Department of Medical Oncology, Strasbourg University Hospitals, Strasbourg, France; h University of Strasbourg, Inserm, Institute for Translational Medicine and Liver Disease (ITM), UMR_1110, Strasbourg, France; i Gastroenterology and Hepatology Service, Strasbourg University Hospitals, Strasbourg, France; j IHU Strasbourg, Strasbourg, France; k Institut Universitaire de France, Paris, France

**Keywords:** Claudin-1 protein, Pancreatic neuroendocrine tumors, Prognostic biomarker, Cell identity

## Abstract

Pancreatic neuroendocrine tumors (pNETs) are biologically heterogeneous neoplasms with variable clinical outcomes. Current prognostic assessment relies largely on mitotic count and Ki-67 index, which may be affected by intratumoral heterogeneity and sampling bias. Additional biomarkers are therefore needed to refine risk stratification and characterize aggressive disease biology. Here, we investigated the prognostic and biological significance of Claudin-1 (CLDN1), a tight junction protein implicated in epithelial tumor progression. CLDN1 expression was assessed by immunohistochemistry in 67 surgically resected pNETs using tissue microarrays. A data-driven cutoff (H-score >50) identified a CLDN1-high subset associated with significantly shorter disease-specific survival (*p* = 0.009) and adverse clinicopathologic features. To define the molecular programs underlying CLDN1 overexpression, we analyzed RNA sequencing profiles from 22 tumors matched for key clinical variables by propensity score matching. CLDN1-high tumors showed attenuation of endocrine lineage markers and enrichment of exocrine and fetal ductal transcriptional programs. Cell-of-origin deconvolution further revealed a shift from endocrine alpha/beta-cell identity toward ductal or acinar-like states. Transcriptomic clustering demonstrated partial convergence between CLDN1-high pNETs and pancreatic ductal adenocarcinoma profiles. In addition, CLDN1-high tumors exhibited activation of epithelial–mesenchymal transition, HIPPO signaling, and immune-related pathways, together with increased stromal and immune cell infiltration. Collectively, our findings identify CLDN1 as a marker of a biologically distinct and clinically aggressive pNETs subset characterized by dedifferentiation, lineage plasticity, and tumor microenvironment remodeling, supporting further evaluation of CLDN1 as a candidate prognostic biomarker and potential therapeutic target in pNETs.

## 1 Introduction

Pancreatic neuroendocrine tumors (pNETs), the second most common form of pancreatic cancer after ductal adenocarcinoma (PDAC), represent a heterogeneous group of epithelial neoplasms arising from pancreatic endocrine cells and exhibiting highly variable clinical outcomes. Approximately half of patients are diagnosed at a metastatic stage, which carries a 5-year survival rate of less than 30% [1]. Prognosis is primarily determined by proliferative activity, as assessed by mitotic count and the Ki-67 proliferation index. The most recent WHO Classification of Tumors of the Digestive System stratifies pNETs into three pathological grades (G1, G2, or G3), which correlate with patient survival 2,3. However, intra-tumoral heterogeneity in proliferative activity can compromise the accuracy of histological grading, particularly when based on limited biopsy material 4,5. These limitations underscore the need for additional biomarkers that can refine prognostic stratification and offer deeper insights into tumor biology beyond proliferation-based parameters.

Among potential candidates, Claudins have emerged as promising biomarkers in several solid tumors [6]. Claudins are members of the tight junction family. In healthy tissues, these transmembrane proteins contribute to cell polarity, paracellular permeability, and epithelial integrity [7]. Dysregulation of Claudin expression, particularly claudin-1 (CLDN1), has been implicated in tumor progression and metastatic behavior across various malignancies 8–11. Meta-analyses across solid tumors further reveal context-dependent roles for CLDN proteins, which can exert tumor-promoting or tumor-suppressive effects depending on the biological context 9,12. In PDAC, CLDN1 is frequently overexpressed compared to normal ductal epithelium and pancreatic intraepithelial neoplasia, and its upregulation has been linked to increased invasiveness, epithelial–mesenchymal transition (EMT), and poor prognosis 13–15.

To date, few studies have examined CLDN1 expression in pNETs or investigated its potential clinical implications. Given the distinct biology of pNETs compared to PDAC, and the emerging role of Claudins in modulating tumor aggressiveness and the tumor microenvironment, and their emergence as therapeutic targets in solid tumors [9], investigating CLDN1 in this context is both timely and relevant.

In this study, we aimed to evaluate the prognostic significance of CLDN1 protein expression in pNETs and to explore its association with tumor biology. We first assessed membranous CLDN1 expression by immunohistochemistry (IHC) and analyzed its correlation with clinical outcomes. We then performed transcriptomic profiling on matched tumor samples to identify gene expression programs associated with CLDN1 expression, including signatures of cellular differentiation, lineage identity, and tumor microenvironment composition.

Our results show that high membranous CLDN1 expression in tumor cells is associated with significantly shorter disease-specific survival (DSS). Moreover, transcriptomic analysis reveals that CLDN1-high pNETs display features of dedifferentiation, a shift toward exocrine lineage identity, and microenvironmental remodeling. These findings suggest that CLDN1 may serve as a clinically relevant prognostic biomarker and reflect a distinct molecular subtype of pNETs with potential therapeutic implications.

## 2 Materials and methods

### 2.1 Ethics statement and patient consent

This retrospective study was approved by the local ethics committee. All patients provided written informed consent for the research use of their clinical data and biological materials. The study was conducted in accordance with applicable institutional and ethical regulations.

### 2.2 Clinical cohorts of pNET

Clinical, pathological and transcriptomic data were collected for 74 patients with pNET who underwent surgical resection at Strasbourg University Hospital between 2007 and 2018 (CHRU cohort) 16. These 74 patients were selected from a previously described cohort of 182 patients based on the availability of high-quality fresh-frozen material 17. No specific exclusion criteria were applied. All histological slides from the time of diagnosis were re-evaluated by two board-certified pathologists (M.-P.C. and A.F.) to confirm the diagnosis of pNET and to reassess tumor grade and stage based on the 5th edition of the WHO Classification of Tumors. Five tissue microarrays (TMAs) were constructed from formalin-fixed paraffin-embedded (FFPE) tumor blocks of 67 cases with available material, derived from our previously reported cohort 16. Each tumor was sampled with four 3-mm cores—two from the tumor center and two from the tumor periphery.

### 2.3 Immunohistochemistry

IHC was performed using rabbit anti–claudin-1 (CLDN1) antibody in the Department of Pathology at the University Hospital of Strasbourg using a Ventana Benchmark ULTRA platform (Ventana Medical Systems, Roche Tissue Diagnostics, Tucson, AZ, USA). Formalin-fixed paraffin-embedded TMA samples were sectioned at five microns and mounted onto positively charged glass slides. A cholangiocarcinoma sample was used as a positive control on each slide. IHC antigen retrieval was carried out with Cell Conditioning 1 (CC1-Ventana) for 36 min. The slides were then incubated with primary antibodies against CLDN1 protein (Elabscience anti-Cldn1 EAB-30939, dilution 1:200) for 32 min at room temperature. Detection was performed using the ultraView Universal DAB detection kit at room temperature, followed by counterstaining with hematoxylin. The staining was assessed by a board-certified pathologist (A.F.) on digitized slides. Immunoreactivity was evaluated using criteria inspired by HER2 immunohistochemical scoring for signal intensity, with a primary focus on tumor cell membranous localization. Both complete circumferential and partial linear membranous staining patterns were considered specific, whereas isolated cytoplasmic staining without membrane reinforcement was interpreted as non-specific background and excluded from analysis. Membranous staining intensity was graded on a four-tier scale (0–3+), adapted from HER2 assessment. A score of 0 corresponded to the absence of membranous staining. A score of 1+ was assigned when membranous staining was not visible at low magnification (×4–×10) and only faintly appreciable at high magnification (×20– ×40). A score of 2+ corresponded to membranous staining weakly visible at low magnification and clearly identifiable at high magnification. A score of 3+ was defined by strong membranous staining readily visible at low magnification. For each case, the proportion of tumor cells exhibiting membranous staining at each intensity level was recorded. An H-score was then calculated by multiplying the percentage of positively membranous-stained tumor cells by the corresponding intensity score (0–3), providing a semi-quantitative measure of CLDN1 membrane expression in tumoral tissue samples. We selected two CLDN1-high tumors and two CLDN1-low tumors, and performed IHC on whole-slide sections to verify the spatial consistency of CLDN1 expression across tumors.

### 2.4 Propensity score matching

To minimize potential clinical confounders and enable transcriptomic comparison of CLDN1-high versus CLDN1-low tumors, propensity score matching (PSM) was performed using the MatchIt R package (v4.5.5). Propensity scores were estimated via logistic regression with CLDN1 status as the outcome and the following clinical covariates as predictors: age, sex, pathological stage, tumor grade, neoadjuvant treatment, type of surgery, functional status, and Ki-67 index category. A 1:1 nearest-neighbor matching approach with a probit link function was applied using a generalized linear model framework, resulting in 11 matched pairs (n = 22) selected from our recently described transcriptomic profiles of our pNET cohort 16.

### 2.5 RNA extraction and sequencing

Total RNA from fresh-frozen samples was converted into sequencing libraries using the NEBNext® UltraTM RNA Library Prep Kit for Illumina® (NEB, USA) following the manufacturer’s recommendations, as recently described 16. To select cDNA fragments of preferentially 150~200 bp in length, the library fragments were purified with the AMPure XP system (Beckman Coulter, Beverly, USA). Then 3 μl USER Enzyme (NEB, USA) was used with size-selected, adaptor-ligated cDNA at 37 °C for 15 min followed by 5 min at 95 °C before PCR. Then PCR was performed with Phusion High-Fidelity DNA polymerase, Universal PCR primers and Index (X) Primer. At last, PCR products were purified (AMPure XP system) and library quality was assessed on the Agilent Bioanalyzer 2100 system. The library preparations were sequenced on an Illumina platform and paired-end reads were generated. Raw FASTQ reads were quality-filtered and trimmed using fastp (v0.23.1). The GRCh38 reference genome and corresponding gene annotation (Ensembl release 109) were downloaded from Ensembl. Clean paired-end reads were aligned to the GRCh38/hg38 human reference genome using STAR (v2.7.10b), and read counts per gene were obtained with FeatureCounts (v1.6.2) [18]. Expression levels were calculated as fragments per kilobase of exon per million mapped reads and subsequently converted to transcripts per million (TPM). A subset of 22 tumor samples was selected for analysis using PSM to balance clinical characteristics between CLDN1-high and CLDN1-low tumors.

### 2.6 Differential expression and functional enrichment analyses

Differential gene expression analysis was conducted using the limma package (v3.54.0) [19]. Genes were considered significantly differentially expressed if they met the following criteria: a nominal *p*-value <0.05, a false discovery rate (FDR) < 0.25, and a fold-change threshold of >1.5 for upregulated genes or <1/1.5 for downregulated genes. For gene set enrichment analysis (GSEA), genes were ranked by log_2_fold-change and analyzed using the clusterProfiler package (v4.6.0) against curated gene sets from the Molecular Signatures Database 20,21.

### 2.7 Estimation of tumor microenvironment composition

The relative abundance of immune and stromal cell populations infiltrating each tumor sample was estimated using the R package MCP-counter (v1.2.0), based on normalized gene expression data [22]. Tumor purity for each bulk RNA-seq sample was assessed using the ESTIMATE R package (1.0.13) [23].

### 2.8 Calculation of oncogenic gene set enrichment score

Ten oncogenic pathway signatures were retrieved from the literature 24,25. Enrichment scores were quantified using single-sample GSEA implemented in the GSVA R package (v1.46.0) [26].

### 2.9 Cell identity prediction

To infer potential cell identities from transcriptomic data, gene expression signature–based classification was performed using nearest template prediction (NTP), implemented via the MOVICS R package (v0.99.17) 27,28. Four major pancreatic cell-type signatures—alpha, beta, acinar, and ductal—were obtained from the Muraro et al. dataset [29]. Prior to NTP analysis, transcriptomic profiles were log_2_-transformed and mean-centered across genes.

### 2.10 Transcriptomic similarity analysis with pancreatic ductal adenocarcinoma

To assess transcriptomic similarity between pNET and PDAC, we retrieved RNA-seq data for 152 PDAC samples from The Cancer Genome Atlas (TCGA) [30]. To balance sample size and account for the generally low tumor purity of PDAC, we selected only those samples with an estimated tumor purity >80%, resulting in a final cohort of 27 PDAC cases. Expression matrices from the 22 matched pNETs samples and the 27 high-purity PDAC samples were then merged. Batch effects were corrected using the sva R package (v3.46.0). To evaluate global transcriptomic similarity, unsupervised hierarchical clustering was performed based on the top 10% most variable genes (as ranked by standard deviation). Clustering was conducted using Euclidean distance and Ward’s linkage method to define sample groupings within the dendrogram.

### 2.11 Statistical analyses

All statistical analyses were performed using R (v4.2.2). Fisher’s exact test was used for categorical variables, and the two-sample Man-n–Whitney *U* test was used for continuous variables. Correlation analyses were performed using Spearman’s rank correlation. Survival analysis was conducted using Kaplan–Meier curves and log-rank tests with the survival R package (v3.4.0) [31]. Hazard ratios (HRs) and 95% confidence intervals (CIs) were estimated using Cox proportional hazards regression. To determine the optimal cutoff for CLDN1 expression, a series of log-rank tests was performed across a range of predefined thresholds (0–25 in steps of 5, then 50–200 in steps of 25), and the threshold yielding the lowest *p*-value was selected. Time-dependent receiver operating characteristic (ROC) analysis was conducted using the timeROC package (v0.4), with 10-year DSS as the outcome. DSS was defined as the time from surgery to death directly attributable to pNET, with deaths from other causes treated as censored events. For unadjusted comparisons, a two-tailed *p* < 0.05 was considered statistically significant.

## 3 Results

### 3.1 Patient cohort and clinicopathological characteristics

The study cohort comprised 67 patients with pNETs, derived from our previously described transcriptomic cohort of 74 pNETs 16, who underwent surgical resection and for whom adequate FFPE material was available for TMA construction and IHC analysis for CLDN1 (Table 1). The median age at diagnosis was balanced across the cohort, with 37 patients (55.2%) younger than 60 years and 30 patients (44.8%) aged 60 years or older. Forty-one patients (61.2%) were male and 26 (38.8%) were female. Most tumors were locally advanced at diagnosis. Pathological T stage was distributed as follows: T1 in 15 cases (22.4%), T2 in 17 cases (25.4%), T3 in 32 cases (47.8%), and T4 in 3 cases (4.5%). Lymph-node involvement was present in 31 patients (46.3%), while distant metastases at diagnosis were observed in 20 patients (29.9%). Accordingly, 29.9% of tumors were classified as stage IV, with the remaining cases distributed across stages I–III. Regarding histological grade, the majority of tumors were well differentiated, with 19 grade 1 tumors (28.8%) and 43 grade 2 tumors (65.2%). Grade 3 tumors accounted for a small subset (n = 4, 6.1%). Ki-67 index distribution was consistent with this grading profile, with 34.8% of tumors in the ≤2% category, 62.1% in the 2–20% category, and 3.0% exceeding 20%. Five patients (7.5%) received neoadjuvant treatment prior to surgery. Surgical management consisted predominantly of standard resections (S0) in 50 cases (74.6%), with more extensive surgical procedures (S1 or S2) performed in the remaining patients. Functional tumors were identified in 11 cases (16.7%), while the majority of tumors were non-functional (83.3%). At last follow-up, disease-specific death had occurred in 13 patients (19.4%), whereas 54 patients (80.6%) were censored for DSS.

### 3.2 CLDN1 protein expression is heterogeneous and prognostic in pNET

**Fig. 1:**
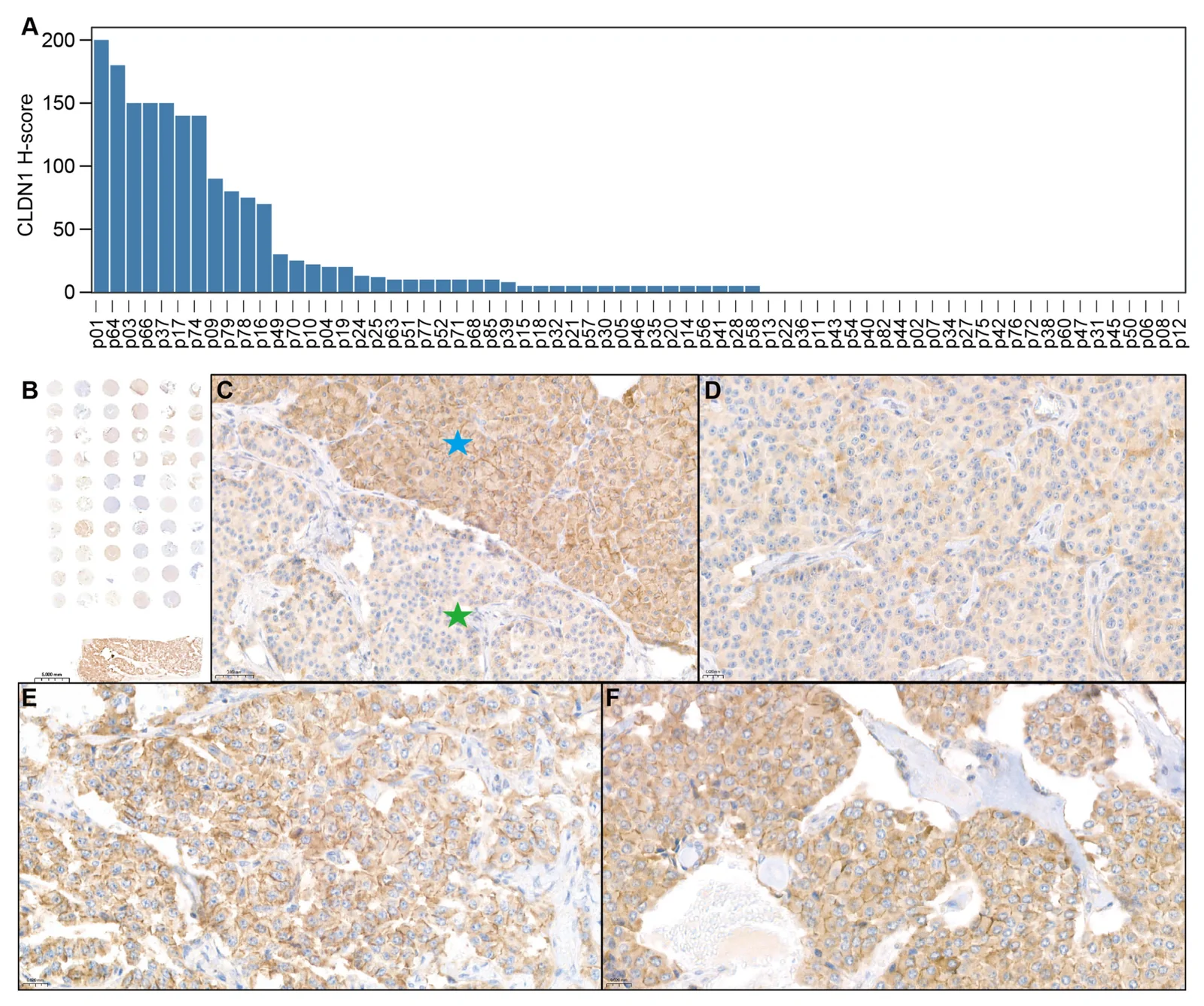
Immunohistochemical assessment of CLDN1 expression in pancreatic neuroendocrine tumors. (A) Distribution of CLDN1 membranous expression in the pNET cohort (n = 67), quantified by H-score. CLDN1 expression shows a right-skewed distribution, with most tumors displaying low or absent staining and a minority exhibiting high expression levels. (B) Low-magnification overview of the tissue microarray (TMA) immunostained with anti–claudin-1 antibody (CLDN1). The TMA was constructed from 67 pancreatic neuroendocrine tumors, with four 3-mm cores sampled from the primary pancreatic lesion for each case. Heterogeneous CLDN1 staining across tumor cores is readily appreciable at low magnification (×0.2). (C) High-power view illustrating absence of membranous CLDN1 staining in tumor cells from patient p75 (green star, ×30). In contrast, adjacent acinar cells show cytoplasmic background signal associated with a delicate, faint, and incomplete membranous staining (blue star, ×30). (D) Higher magnification of the same CLDN1-low tumor (patient p75, ×40), confirming the lack of membranous CLDN1 expression in tumor cells. (E–F) Representative high-power images (×40) from CLDN1-expressing tumors showing moderate (2+), usually incomplete membranous staining in the majority of tumor cells, illustrated by patient p64 (E) and patient p66 (F). Nuclei are counterstained with hematoxylin.

CLDN1 expression, quantified by H-score, ranged from 0 to 200 (median = 5; interquartile range = 0–75), with a right-skewed distribution: 60% of tumors had low or undetectable levels (H-score ≤10), while a minority showed high expression (H-score ≥150) (Fig. 1A and B). Most tumors showed absent or minimal membranous Claudin-1 expression, with no staining or only weak membranous immunoreactivity (1+) restricted to rare tumor cells (<10%), resulting in low H-scores (<10) (Fig. 1C and D). In contrast, a distinct subset of tumors exhibited membranous Claudin-1 expression, generally incomplete, with moderate or, less frequently, strong intensity involving a large proportion of tumor cells (Fig. 1E and F). CLDN1 expression was generally homogeneous across the different cores from the same patient. Weak incomplete membranous staining was also observed in normal pancreatic acinar and ductal cells. Occasional cytoplasmic staining was detected in both tumor and non-tumor cells and was considered non-specific and therefore not scored. Whole-slide evaluation revealed a spatially consistent membranous staining pattern across tumor cells.

**Fig. 2:**
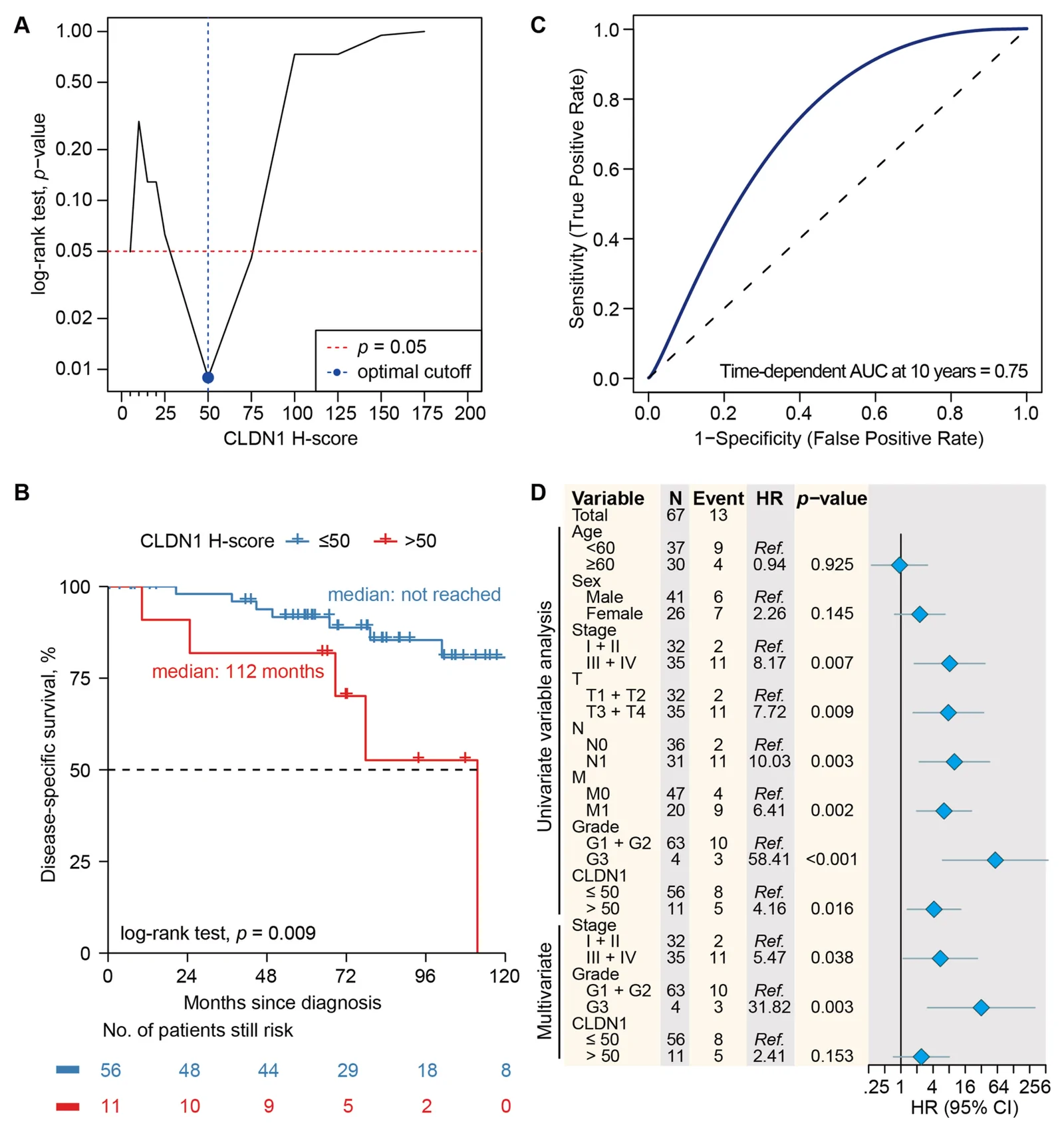
Determination of an optimal CLDN1 H-score cutoff and association with disease-specific survival in pNETs. (A) Determination of the optimal CLDN1 H-score cutoff for prognostic stratification. Log-rank test *p*-values are plotted across a range of predefined CLDN1 H-score thresholds. The dashed red horizontal line indicates the nominal significance level (*p* = 0.05). The dashed blue vertical line and blue dot indicate the cutoff yielding the minimum log-rank *p*-value (H-score = 50), which was selected for subsequent analyses. (B) Kaplan–Meier analysis of disease-specific survival (DSS) according to CLDN1 expression status, stratified using the H-score cutoff of 50. Tick marks indicate censored observations, and numbers at risk are shown below the plot. (C) Time-dependent receiver operating characteristic (ROC) curve evaluating the performance of CLDN1 H-score for predicting 10-year disease-specific survival. The dashed diagonal line represents random classification. (D) Forest plot summarizing univariate and multivariate Cox proportional hazards analyses for disease-specific survival. Variables evaluated include age, sex, tumor stage, T stage, nodal status, metastatic status, tumor grade, and CLDN1 expression. Hazard ratios (HRs) and 95% confidence intervals (CIs) are shown.

To determine a clinically meaningful cutoff for prognostication, we applied a H-score escalation with log-rank test using Kaplan–Meier survival estimates. A threshold of H-score >50 yielded the most significant stratification of DSS (*p* = 0.009) and corresponded to the minimum log-rank *p*-value (Fig. 2A). Patients with high CLDN1 expression (n = 11) had a median DSS of 112 months, whereas the median was not reached for the low CLDN1 group (n = 56) (Fig. 2B). Time-dependent ROC analysis at 10 years further supported this cutoff, identifying an optimal threshold of 50 based on the Youden index (sensitivity = 0.79, specificity = 0.55), with an AUC of 0.73 (Fig. 2C). Based on these findings, tumors were classified into CLDN1-high (>50) and CLDN1-low (≤ 50) groups for downstream analysis.

Among the 11 CLDN1-high tumors, two were classified as grade 3 (18%), representing a five-fold enrichment compared to the low CLDN1 group (3.6%), although this difference did not reach statistical significance (*p* = 0.152; Table 2). In univariate Cox regression analysis, the high CLDN1 group was associated with an increased risk of cancer-specific mortality (HR = 4.17; 95% CI, 1.31–13.28, *p* = 0.016), along with other clinical factors such as TNM stage and tumor grade (Fig. 2D). However, in the multivariable model adjusting for tumor stage and grade, CLDN1 protein expression was not retained as an independent prognostic factor, suggesting that its prognostic impact may be confounded by co-linearity with other markers of aggressiveness.

### 3.3 Transcriptomic profiling after propensity score matching reveals CLDN1-associated molecular signatures

To reduce clinical confounding and isolate the transcriptional impact of CLDN1 protein expression, we employed PSM to select 11 CLDN1-high and 11 CLDN1-low tumors, balanced across clinical variables including age, sex, stage, grade, Ki-67 index, neoadjuvant treatment, surgery type, and functional status (all *p* > 0.3; Table 2). This matching strategy preserved sample size and enhanced comparability, allowing the analysis of CLDN1-associated molecular alterations within a clinically high-risk pNET subset.

**Fig. 3:**
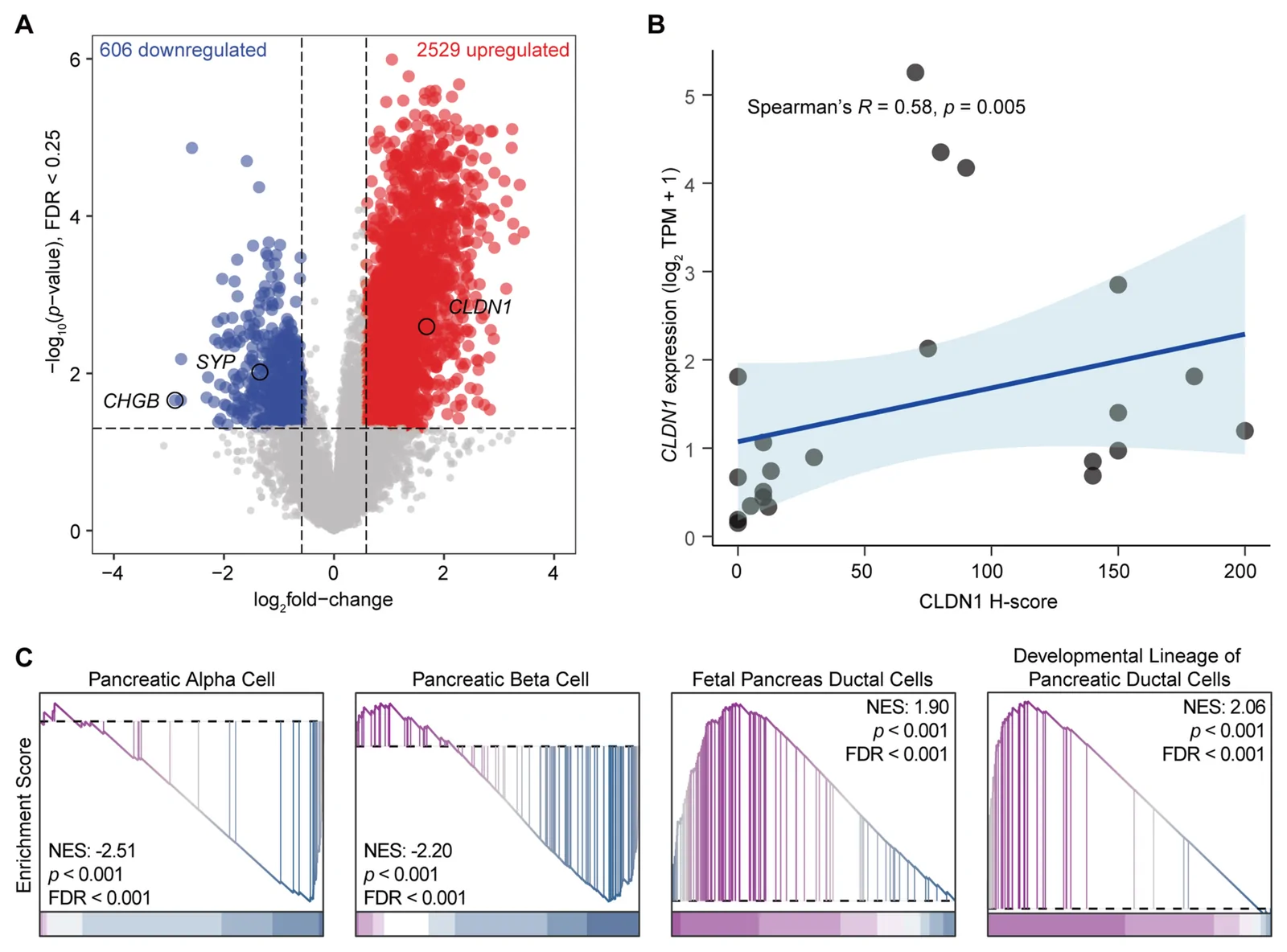
Transcriptomic programs associated with high CLDN1 protein expression in pNETs. (A) Volcano plot showing differential gene expression between CLDN1-high (H-score >50) and CLDN1-low (H-score ≤50) tumors in the propensity score–matched RNA-seq cohort (n = 22; 11 vs 11). Genes are plotted according to log_2_ fold change and –log_10_(*p*-value) (FDR <0.25). Representative genes are highlighted. (B) Spearman correlation between CLDN1 protein expression (H-score) and *CLDN1* transcript abundance (log_2_[TPM + 1]) across matched tumors. Shaded area indicates the 95% confidence interval of the regression fit. (C) Gene set enrichment analysis (GSEA) illustrating lineage-associated transcriptional programs in CLDN1-high tumors. Normalized enrichment scores (NES), nominal *p*-values, and false discovery rates (FDR) are indicated for each gene set.

Differential expression analysis identified 2529 genes upregulated and 606 genes downregulated in the CLDN1-high group (Fig. 3A). As expected, *CLDN1* transcript levels were significantly elevated in this group (fold change = 3.2; *p* = 0.003; FDR = 0.042), with a strong correlation between transcript and protein levels (Spearman’s *R* = 0.58, *p* = 0.005; Fig. 3B). Notably, several neuroendocrine markers (e.g., *CHGB, SYP*) were markedly downregulated in CLDN1-high tumors (Fig. 3A). GSEA further supported a shift in differentiation status: pathways associated with beta-cell and alpha-cell identity were significantly downregulated, while gene sets linked to fetal ductal and exocrine lineage programs were upregulated in the CLDN1-high group (Fig. 3C).

### 3.4 High CLDN1 pNETs exhibit a shift toward exocrine cell identity

To further evaluate the observed loss of endocrine features, we inferred cell-of-origin identity across four pancreatic lineages: alpha, beta, acinar, and ductal. Among CLDN1-high tumors, 7 of 11 (64%) were predicted to derive from exocrine lineages (4 ductal, 3 acinar), whereas all 11 CLDN1-low tumors were classified as endocrine-derived (9 alpha, 2 beta) (*p* = 0.004).

**Fig. 4:**
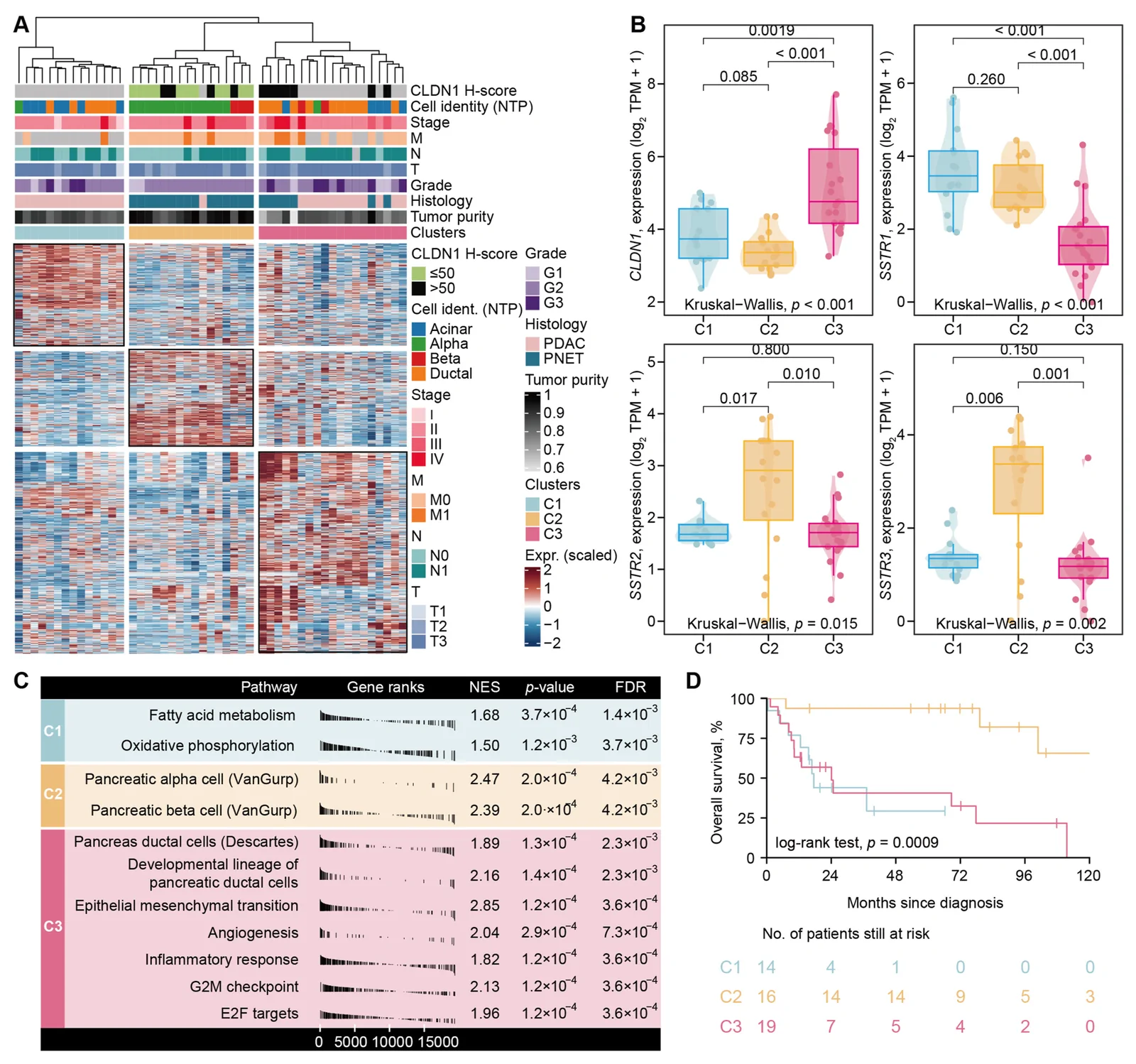
Transcriptomic clustering reveals lineage- and outcome-associated pNET subgroups with partial convergence toward PDAC. (A) Unsupervised hierarchical clustering of propensity score–matched pNET samples (n = 22) together with high-purity TCGA pancreatic ductal adenocarcinoma (PDAC) samples (n = 27), based on the top 10% most variable genes. The resulting dendrogram identifies three transcriptional clusters (C1–C3). Clinical annotations are shown above the heatmap, including CLDN1 H-score, inferred cell identity by nearest template prediction (NTP), TNM stage, tumor grade, histology (pNET vs PDAC), tumor purity, and cluster assignment. CLDN1-high (H-score >50) pNETs preferentially localize to the mixed C3 cluster, which also contains a subset of PDAC samples. (B) Transcriptomic expression of *CLDN1* and somatostatin receptor genes (*SSTR1*–*3*) across transcriptional clusters. Boxplots display log_2_(TPM + 1) expression values, with individual samples overlaid. Kruskal–Wallis tests indicate significant differences across clusters, and pairwise comparisons by two-sample Man-n–Whitney *U* test are shown. (C) Gene set enrichment analysis (GSEA) highlighting dominant biological programs associated with each cluster. Normalized enrichment scores (NES), *p*-values, and FDR values are shown. (D) Kaplan–Meier analysis of overall survival according to transcriptomic cluster assignment. Tick marks indicate censored observations, and numbers at risk are shown below the plot.

To validate this shift, we performed unsupervised clustering on transcriptomes from the 22 matched pNETs alongside 27 high-purity PDAC samples (Fig. 4A). Clustering based on highly variable genes identified three transcriptional groups: C1 (n = 14; all PDACs), C2 (n = 16; 15 pNETs and 1 PDAC), and C3 (n = 19; a mix of 7 pNETs and 12 PDACs). Notably, all 7 pNETs within the mixed C3 cluster belonged to the CLDN1-high group (100%), whereas 11 of 15 pNETs in the pNETs-enriched C2 cluster were CLDN1-low (73%) (*p* = 0.004). These findings indicate that CLDN1-high pNETs share transcriptional features with a subset of PDAC, suggesting a partial phenotypic convergence.

Of note, *CLDN1* expression at the RNA level was highest in C3, followed by C1, whereas C2 exhibited low *CLDN1* expression (*p* < 0.001; Fig. 4B). In addition to *CLDN1*, tumors in C3 showed downregulation of somatostatin receptor genes (*SSTR1, SSTR2*, and *SSTR3*), accompanied by upregulation of gene sets associated with fetal ductal and exocrine lineage programs (Fig. 4B). GSEA further demonstrated that C3 was enriched for multiple HALLMARK pathways, including EMT, angiogenesis, immune-related, and proliferative programs. In contrast, C2 showed preferential enrichment of the pancreatic beta-cell HALL-MARK signature, while C1 was characterized by activation of metabolic pathways, including fatty acid metabolism and oxidative phosphorylation (Fig. 4C). Because DSS data were largely unavailable for TCGA PDAC samples, we used overall survival as a surrogate endpoint. This analysis showed that C2 had the most favorable outcomes, whereas C1 and C3 showed similarly poor outcomes (*p* = 0.0009; Fig. 4D).

### 3.5 Oncogenic pathway activation and tumor microenvironment remodeling in high CLDN1 pNETs

**Fig. 5:**
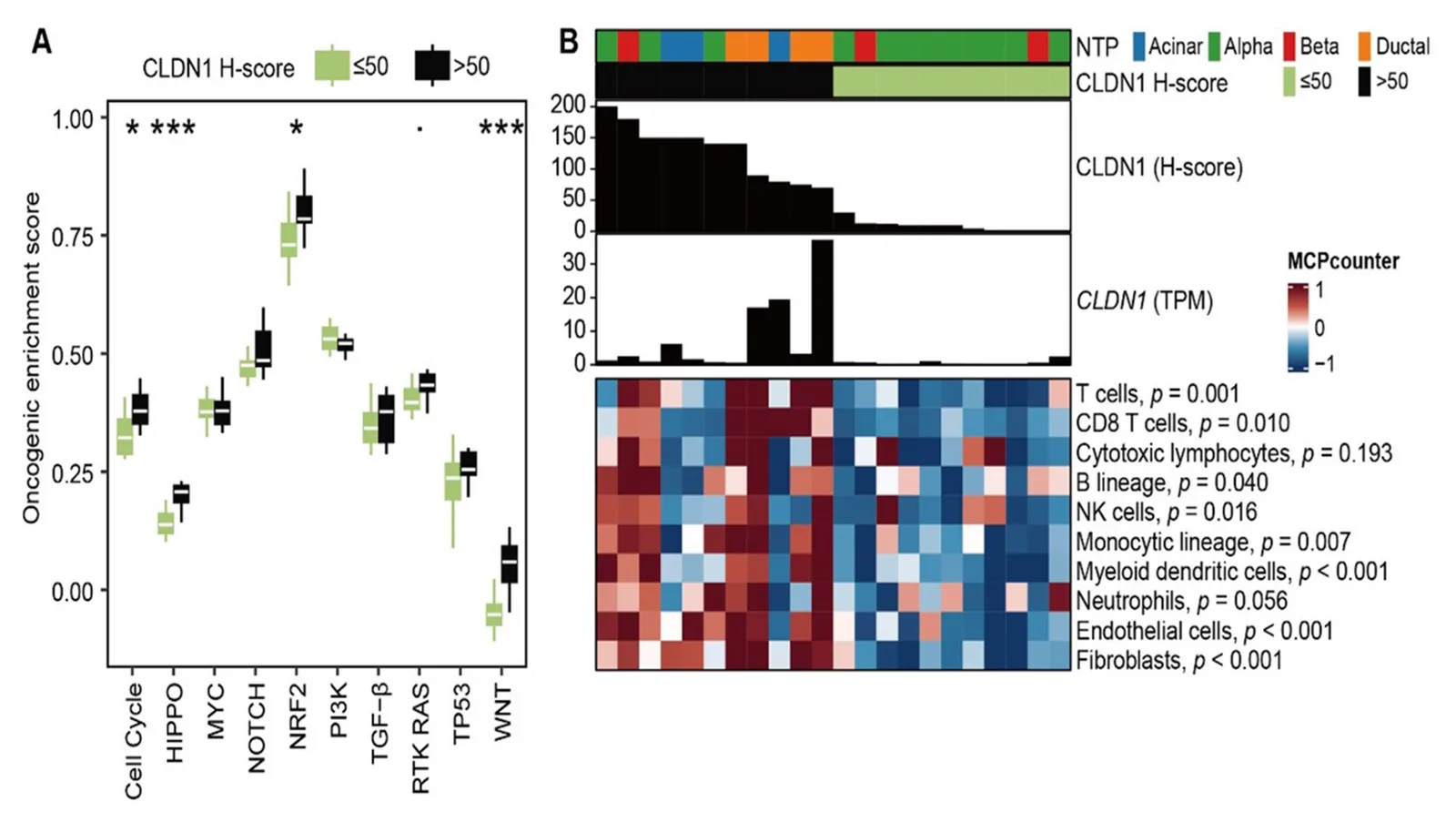
Oncogenic pathway activation and tumor microenvironment remodeling associated with high CLDN1 protein expression in pNETs. (A) Comparison of oncogenic pathway enrichment scores between CLDN1-low (H-score ≤50) and CLDN1-high (H-score >50) pNETs in the propensity score–matched cohort. Enrichment scores were calculated using single-sample gene set enrichment analysis. Boxplots display median and interquartile range. Statistical significance was assessed using the two-sample Mann–Whitney *U* test (.*p* < 0.1; *p < 0.05; **p < 0.001). (B) Tumor microenvironment composition associated with CLDN1 expression. Upper panels show sample-wise CLDN1 H-scores and corresponding *CLDN1* Mrna expression (TPM), annotated with inferred cell identity by nearest template prediction (NTP). The heatmap depicts relative abundance scores of immune and stromal cell populations estimated using MCP-counter, scaled across samples. *P*-values by two-sample Mann–Whitney *U* test indicate comparisons between CLDN1-high and CLDN1-low groups.

Given the poor prognosis observed in the CLDN1-high group, we explored its association with oncogenic signaling. Among ten canonical pathways assessed, CLDN1-high tumors showed significant upregulation of cell cycle, HIPPO, NRF2, and WNT pathways (all *p* < 0.05; Fig. 5A). As these pathways are known to shape the tumor microenvironment (TME), we next applied MCP-counter to estimate TME composition. This analysis revealed that CLDN1-high tumors were significantly enriched in both immune components (T cells, B cells) and stromal compartments (fibroblasts, endothelial cells) (Fig. 5B). Collectively, these findings suggest that high CLDN1 expression defines a biologically aggressive pNET subtype marked by dedifferentiation, stromal remodeling, and immune infiltration.

## 4 Discussion

PNETs encompass a heterogeneous spectrum of neoplasms characterized by highly variable clinical behavior with respect to local invasion and metastatic potential. Current histological approaches provide only imperfect prognostic stratification, a limitation that is especially pronounced in biopsy material, and further studies are needed to better understand the cellular mechanisms and tumorigenic pathways underlying these pancreatic tumors.

Tight junctions are intercellular protein complexes that maintain tissue homeostasis and integrity by regulating paracellular permeability and preserving cell polarity [7]. Among the tight junction protein family members, Claudins have recently been recognized as bidirectional signaling hubs, modulating differentiation, growth, and proliferation, as well as regulating cell migration and motility [6,32]. In cancer, altered Claudin expression is frequently observed, disrupting normal cellular machinery and compromising the maintenance of homeostasis [9]. Claudin overexpression can contribute to uncontrolled growth and enhanced invasive potential [33]. CLDN1, a member of the claudin family first identified in 1998 [34], has attracted particular attention both as a driver and therapeutic target for solid tumors. CLDN1 overexpression has been associated with increased invasiveness and metastatic potential in several malignancies, including liver cancer, gastric carcinoma and colorectal cancer 9,35–39. Mechanistically, CLDN1 has been shown to promote oncogenic signaling, stemness and reprogramming of the tumor microenvironment in hepatocellular carcinoma, underscoring its role in driving tumor progression and cellular plasticity [8,35,40].

Although CLDN1 has been extensively studied in other epithelial malignancies, including PDAC [13,41], its role in pNETs remains poorly investigated. Only one previous study has investigated CLDN1 immunohistochemical expression in pNETs. Borka et al. reported no significant expression of CLDN1 in a cohort of 20 tumors, in contrast to the findings of our study [42]. Several factors may account for this discrepancy. First, the relatively small sample size of the Borka et al. cohort may have limited the ability to detect biologically relevant subsets. Second, the tumors included in their study were likely less aggressive than those represented in our cohort, where CLDN1-high cases were enriched in grade 3 neoplasms and associated with adverse clinicopathological features. Taken together, these differences suggest that CLDN1 expression may be specifically upregulated in more aggressive pNET subsets, which were underrepresented in earlier series.

In our study, both complete and partial membranous CLDN1 immunoreactivity were considered biologically meaningful, whereas cytoplasmic staining was deliberately excluded from analysis. Although several studies have reported the presence of cytoplasmic CLDN1 immunoreactivity in different tumor types, the biological significance of this pattern remains unclear and its interpretation is highly variable across studies [43]. Given that Claudin-1 mediates oncogenic signaling [9], with a primary functional role at the cell membrane [44] and cell membrane expressed CLDN1 is a target for therapeutic monoclonal antibodies [9,36], we chose to focus exclusively on membranous localization as the most biologically relevant and technically robust readout of expression. This approach is consistent with the established biology of claudin family proteins and with immunohistochemical studies emphasizing membrane-associated Claudin-1 as the relevant indicator of expression. Moreover, membranous scoring strategies analogous to those routinely applied to other therapeutically relevant cell-surface proteins, such as HER2, could contribute to improved reproducibility and clinical interpretability in immunohistochemical analyses. Accordingly, by focusing on membrane-associated Claudin-1 expression, our scoring strategy was designed to capture biologically and potentially therapeutically relevant protein localization while minimizing noise related to non-specific or ambiguously localized intracellular staining.

In this study, we identified CLDN1 as a novel prognostic marker in pNETs, with high membranous expression associated with shorter DSS. Through integrative transcriptomic analyses, we demonstrated that CLDN1-high tumors exhibit features of dedifferentiation, a shift toward exocrine lineage identity, and substantial remodeling of the tumor microenvironment. These findings support the relevance of CLDN1 as both a surrogate of aggressive tumor biology and a potential biomarker for risk stratification in pNETs.

Our data suggest that in the context of pNETs, high CLDN1 expression may signify a divergence from classical endocrine differentiation programs toward exocrine and ductal phenotypes. This is supported by transcriptomic downregulation of alpha- and beta-cell identity genes and upregulation of fetal ductal markers in CLDN1-high tumors. Furthermore, the use of Nearest Template Prediction revealed that a majority of CLDN1-high tumors transcriptionally resemble acinar or ductal lineages—an identity rarely observed in well-differentiated neuroendocrine tumors.

This lineage shift is not merely a molecular curiosity but appears to have clinical implications. CLDN1-high tumors were overrepresented among grade 3 neoplasms and clustered with PDAC samples in unsupervised transcriptomic analyses. These tumors also displayed enrichment in oncogenic pathways such as HIPPO signaling and EMT, both of which have been implicated in tumor progression and therapeutic resistance. Taken together, these observations suggest that CLDN1 expression may define a dedifferentiated and potentially more plastic pNET subtype, with implications for both prognosis and therapeutic targeting.

The association of CLDN1 with an altered tumor microenvironment is another key finding of our study. CLDN1-high tumors showed increased infiltration by immune and stromal cell populations, including T cells, NK cells, neutrophils, fibroblasts, and endothelial cells. This enrichment suggests that CLDN1 expression may shape, or be shaped by, microenvironmental signals that promote immune evasion or tumor-supportive stroma. While further work is needed to determine whether this immune-rich phenotype reflects an active anti-tumor response or an immunosuppressive milieu, the finding opens avenues for immunotherapeutic exploration in this subset of tumors.

Interestingly, CLDN1 expression was not an independent prognostic factor in multivariable analysis, likely due to its association with known adverse features such as high grade. Nonetheless, the transcriptomic and microenvironmental distinctiveness of CLDN1-high tumors supports its role as a marker of biological aggressiveness, beyond what is captured by current histopathological grading. In the clinical setting, CLDN1 IHC could thus complement Ki-67 and mitotic count to improve prognostication—especially in biopsies with limited material or ambiguous proliferation indices.

In addition to its prognostic value, the present study highlights Claudin-1 as a potential therapeutic target in pancreatic neuroendocrine tumors. The membranous localization and overexpression observed in aggressive pNETs provide a conceptual framework for targeted approaches such as antibody–drug conjugates (ADCs), which exploit cell-surface antigens for selective drug delivery. Claudin-1 has recently gained attention as a target for antibody-drug conjugates (ADCs) in other epithelial cancers (NCT06747585), supporting the translational relevance of our findings [9,36,45]. Thus, Claudin-1 IHC may ultimately serve not only as a biomarker of outcome, but also as a predictive marker to guide patient selection for future Claudin-1-directed therapies. Further functional and preclinical studies will be required to validate its suitability as a companion diagnostic in pNETs.

From a broader perspective, our findings align with the emerging view that pNETs encompass transcriptionally and biologically diverse subtypes, potentially reflecting distinct cells of origin or trajectories of tumor evolution [46]. The partial convergence of CLDN1-high pNETs with PDAC-like features raises the hypothesis that some pNETs may adopt ductal programs during progression, challenging the traditional dichotomy between endocrine and exocrine pancreatic tumors.

Although we used propensity score matching to reduce clinical confounding, the sample size of the transcriptomic cohort was relatively small, and validation in independent cohorts is needed. Moreover, our analyses were based on bulk RNA-seq and TMA analysis, which cannot fully resolve the spatial architecture or cellular heterogeneity of CLDN1-high tumors. Single-cell and spatial transcriptomic approaches would be valuable for dissecting the precise relationship between CLDN1 expression, lineage plasticity, and the tumor microenvironment.

In conclusion, our study demonstrates that high CLDN1 expression identifies a subset of pNETs with adverse clinical outcomes, exocrine lineage reprogramming, and an altered tumor microenvironment. CLDN1 may serve as a surrogate marker of dedifferentiation and stromal activation, highlighting its potential utility in improving risk stratification and uncovering novel therapeutic vulnerabilities in pNETs.

## Figures and Tables

**Table 1:** Clinicopathological characteristics of the pancreatic neuroendocrine tumor cohort stratified by CLDN1 protein expression (n = 67).

Variable	Overall, n = 67	≤50, n = 56	>50, n = 11	*p*-value^a^	Missing^b^
Age, %				0.703	0.0
<60	37 (55.2)	32 (57.1)	5 (45.5)		
≥60	30 (44.8)	24 (42.9)	6 (54.5)		
Sex, %				0.603	0.0
Female	26 (38.8)	23 (41.1)	3 (27.3)		
Male	41 (61.2)	33 (58.9)	8 (72.7)		
T, %				0.124	0.0
T1	15 (22.4)	15 (26.8)	0 (0.0)		
T2	17 (25.4)	12 (21.4)	5 (45.5)		
T3	32 (47.8)	26 (46.4)	6 (54.5)		
T4	3 (4.5)	3 (5.4)	0 (0.0)		
N, %				0.351	0.0
N0	36 (53.7)	32 (57.1)	4 (36.4)		
N1	31 (46.3)	24 (42.9)	7 (63.6)		
M, %				1.000	0.0
M0	47 (70.1)	39 (69.6)	8 (72.7)		
M1	20 (29.9)	17 (30.4)	3 (27.3)		
Stage, %				0.191	0.0
I	15 (22.4)	15 (26.8)	0 (0.0)		
II	17 (25.4)	13 (23.2)	4 (36.4)		
III	15 (22.4)	11 (19.6)	4 (36.4)		
IV	20 (29.9)	17 (30.4)	3 (27.3)		
Grade, %				0.156	1.5
G1	19 (28.8)	17 (30.9)	2 (18.2)		
G2	43 (65.2)	36 (65.5)	7 (63.6)		
G3	4 (6.1)	2 (3.6)	2 (18.2)		
Neoadjuvant,				0.687	0.0
%					
No	62 (92.5)	51 (91.1)	11 (100.0)		
Yes	5 (7.5)	5 (8.9)	0 (0.0)		
Surgery type,				0.336	0.0
%					
S0	50 (74.6)	43 (76.8)	7 (63.6)		
S1	9 (13.4)	6 (10.7)	3 (27.3)		
S2	8 (11.9)	7 (12.5)	1 (9.1)		
Functional, %				0.555	1.5
Yes	11 (16.7)	8 (14.5)	3 (27.3)		
No	55 (83.3)	47 (85.5)	8 (72.7)		
Ki67, %				0.236	1.5
(0,2]	23 (34.8)	21 (38.2)	2 (18.2)		
(2,20]	41 (62.1)	33 (60.0)	8 (72.7)		
(20,100]	2 (3.0)	1 (1.8)	1 (9.1)		
DSS, %				0.049	0.0
Censored	54 (80.6)	48 (85.7)	6 (54.5)		
Dead	13 (19.4)	8 (14.3)	5 (45.5)		

Note: Categorical variables were presented as proportions, with any missing data excluded from the respective calculations.

a *p*-values were calculated using Fisher’s exact test for categorical data.

b Missing rates were presented for each variable where data was unavailable.

**Table 2:** Clinicopathological characteristics of the propensity score–matched pancreatic neuroendocrine tumor cohort used for transcriptomic analysis (n = 22).

Variable	Overall, n = 22	≤50, n = 11	>50, n = 11	*p*-value^a^
Age, %				1.000
<60	10 (45.5)	5 (45.5)	5 (45.5)	
≥60	12 (54.5)	6 (54.5)	6 (54.5)	
Sex, %				1.000
Female	7 (31.8)	4 (36.4)	3 (27.3)	
Male	15 (68.2)	7 (63.6)	8 (72.7)	
T, %				0.658
T2	8 (36.4)	3 (27.3)	5 (45.5)	
T3	14 (63.6)	8 (72.7)	6 (54.5)	
N, %				0.394
N0	11 (50.0)	7 (63.6)	4 (36.4)	
N1	11 (50.0)	4 (36.4)	7 (63.6)	
M, %				0.580
M0	18 (81.8)	10 (90.9)	8 (72.7)	
M1	4 (18.2)	1 (9.1)	3 (27.3)	
Stage, %				0.375
II	11 (50.0)	7 (63.6)	4 (36.4)	
III	7 (31.8)	3 (27.3)	4 (36.4)	
IV	4 (18.2)	1 (9.1)	3 (27.3)	
Grade, %				0.325
G1	4 (18.2)	2 (18.2)	2 (18.2)	
G2	16 (72.7)	9 (81.8)	7 (63.6)	
G3	2 (9.1)	0 (0.0)	2 (18.2)	
Neoadjuvant, %				N/A
No	22 (100.0)	11 (100.0)	11 (100.0)	
Surgery type, %				0.311
S0	17 (77.3)	10 (90.9)	7 (63.6)	
S1	4 (18.2)	1 (9.1)	3 (27.3)	
S2	1 (4.5)	0 (0.0)	1 (9.1)	
Functional, %				0.580
Yes	4 (18.2)	1 (9.1)	3 (27.3)	
No	18 (81.8)	10 (90.9)	8 (72.7)	
Ki67, %				0.589
(0,2]	4 (18.2)	2 (18.2)	2 (18.2)	
(2,20]	17 (77.3)	9 (81.8)	8 (72.7)	
(20,100]	1 (4.5)	0 (0.0)	1 (9.1)	

Note: Categorical variables were presented as proportions, with any missing data excluded from the respective calculations.

a *p*-values were calculated using Fisher’s exact test for categorical data.

## Data Availability

The RNA-seq dataset analyzed in this study comprises 22 pNET tumor samples selected from our previously published cohort 16, and used here for CLDN1-stratified transcriptomic analyses after propensity score matching. Pre-processed RNA-seq expression data from the parent cohort, together with associated clinical sample metadata, are publicly available through Mendeley Data at https://doi.org/10.17632/r9m66rjtxy.1.

## References

[1] Dasari A, Shen C, Halperin D (2017). Trends in the incidence, prevalence, and survival outcomes in patients with neuroendocrine tumors in the United States. JAMA Oncol.

[2] McCall CM, Shi C, Cornish TC (2013). Grading of well-differentiated pancreatic neuroendocrine tumors is improved by the inclusion of both Ki67 proliferative index and mitotic rate. Am J Surg Pathol.

[3] Basturk O, Yang Z, Tang LH (2015). The high-grade (WHO G3) pancreatic neuroendocrine tumor category is morphologically and biologically heterogenous and includes both well differentiated and poorly differentiated neoplasms. Am J Surg Pathol.

[4] Yang Z, Tang LH, Klimstra DS (2011). Effect of tumor heterogeneity on the assessment of Ki67 labeling index in well-differentiated neuroendocrine tumors metastatic to the liver: implications for prognostic stratification. Am J Surg Pathol.

[5] Grillo F, Valle L, Ferone D (2017). KI-67 heterogeneity in well differentiated gastro-entero-pancreatic neuroendocrine tumors: when is biopsy reliable for grade assessment?. Endocrine.

[6] Nehme Z, Roehlen N, Dhawan P, Baumert TF (2023). Tight junction protein signaling and cancer biology. Cells.

[7] Zihni C, Mills C, Matter K, Balda MS (2016). Tight junctions: from simple barriers to multifunctional molecular gates. Nat Rev Mol Cell Biol.

[8] Wu YL, Zhang S, Wang GR, Chen YP (2008). Expression transformation of claudin-1 in the process of gastric adenocarcinoma invasion. World J Gastroenterol.

[9] Saviano A, Toso A, Klempner SJ, Baumert TF (2026). Claudin proteins as emerging therapeutic targets for solid tumours. Nat Rev Cancer.

[10] Dhawan P, Singh AB, Deane NG (2005). Claudin-1 regulates cellular transformation and metastatic behavior in colon cancer. J Clin Investig.

[11] Akasaka H, Sato F, Morohashi S (2010). Anti-apoptotic effect of claudin-1 in tamoxifen-treated human breast cancer MCF-7 cells. BMC Cancer.

[12] Li J (2021). Context-dependent roles of claudins in tumorigenesis. Front Oncol.

[13] Kyuno D, Asano H, Okumura R (2025). The role of Claudin-1 in enhancing pancreatic cancer aggressiveness and drug resistance via metabolic pathway modulation. Cancers.

[14] Tsukahara M, Nagai H, Kamiakito T (2005). Distinct expression patterns of claudin-1 and claudin-4 in intraductal papillary-mucinous tumors of the pancreas. Pathol Int.

[15] Kondo J, Sato F, Kusumi T (2008). Claudin-1 expression is induced by tumor necrosis factor-alpha in human pancreatic cancer cells. Int J Mol Med.

[16] Lu X, Baltzinger P, Xu L (2026). Molecular taxonomy of pancreatic neuroendocrine tumors reveals BEND2-fusions-driven transcriptional plasticity and therapeutic vulnerabilities. Cell Rep Med.

[17] Debien V, Davidson G, Baltzinger P (2021). Involvement of Neutrophils in Metastatic Evolution of Pancreatic Neuroendocrine Tumors. Cancers.

[18] Liao Y, Smyth GK, Shi W (2013). featureCounts: an efficient general purpose program for assigning sequence reads to genomic features. Bioinformatics.

[19] Ritchie ME, Phipson B, Wu D (2015). Limma powers differential expression analyses for RNA-sequencing and microarray studies. Nucleic Acids Res.

[20] Wu T, Hu E, Xu S (2021). clusterProfiler 4.0: A universal enrichment tool for interpreting omics data. Innovation.

[21] Liberzon A, Subramanian A, Pinchback R, Thorvaldsdóttir H, Tamayo P, Mesirov JP (2011). Molecular signatures database (MSigDB) 3.0. Bioinformatics.

[22] Becht E, Giraldo NA, Lacroix L (2016). Estimating the population abundance of tissue-infiltrating immune and stromal cell populations using gene expression. Genome Biol.

[23] Yoshihara K, Shahmoradgoli M, Martínez E (2013). Inferring tumour purity and stromal and immune cell admixture from expression data. Nat Commun.

[24] Sanchez-Vega F, Mina M, Armenia J (2018). Oncogenic Signaling Pathways in The Cancer Genome Atlas. Cell.

[25] Su X, Lu X, Bazai Sk (2023). Delineating the interplay between oncogenic pathways and immunity in anaplastic Wilms tumors. Nat Commun.

[26] Hänzelmann S, Castelo R, Guinney J (2013). GSVA: gene set variation analysis for microarray and RNA-seq data. BMC Bioinf.

[27] Lu X, Meng J, Zhou Y, Jiang L, Yan F (2020). MOVICS: an R package for multi-omics integration and visualization in cancer subtyping. Bioinformatics.

[28] Zhu J, Zhu Y, Wang X (2024). MOVICShiny: An interactive website for multi-omics integration and visualisation in cancer subtyping. Clin Transl Med.

[29] Muraro MJ, Dharmadhikari G, Grün D (2016). A single-cell transcriptome atlas of the human pancreas. Cell Syst.

[30] Peran I, Madhavan S, Byers SW, McCoy MD (2018). Curation of the pancreatic ductal adenocarcinoma subset of the cancer genome atlas is essential for accurate conclusions about survival-related molecular mechanisms. Clin Cancer Res.

[31] Lu X, Li K, Li Z (2025). FigureYa: a standardized visualization framework for enhancing biomedical data interpretation and research efficiency. iMeta.

[32] Martin TA (2014). The role of tight junctions in cancer metastasis. Semin Cell Dev Biol.

[33] Förster C (2008). Tight junctions and the modulation of barrier function in disease. Histochem Cell Biol.

[34] Furuse M, Fujita K, Hiiragi T, Fujimoto K, Tsukita S (1998). Claudin-1 and -2: novel integral membrane proteins localizing at tight junctions with no sequence similarity to occludin. J Cell Biol.

[35] Kinugasa T, Akagi Y, Ochi T (2012). Increased claudin-1 protein expression in hepatic metastatic lesions of colorectal cancer. Anticancer Res.

[36] Roehlen N, Muller M, Nehme Z (2023). Treatment of HCC with claudin-1-specific antibodies suppresses carcinogenic signaling and reprograms the tumor microenvironment. J Hepatol.

[37] Huang J, Zhang L, He C (2015). Claudin-1 enhances tumor proliferation and metastasis by regulating cell anoikis in gastric cancer. Oncotarget.

[38] Roehlen N, Saviano A, El Saghire H (2022). A monoclonal antibody targeting nonjunctional claudin-1 inhibits fibrosis in patient-derived models by modulating cell plasticity. Sci Transl Med.

[39] Zeisel MB, Dhawan P, Baumert TF (2019). Tight junction proteins in gastrointestinal and liver disease. Gut.

[40] Suh Y, Yoon CH, Kim RK (2013). Claudin-1 induces epithelial-mesenchymal transition through activation of the c-Abl-ERK signaling pathway in human liver cells. Oncogene.

[41] Alikanoglu AS, Gunduz S, Demirpence O (2015). Expression pattern and prognostic significance of claudin 1, 4 and 7 in pancreatic cancer. Asian Pac J Cancer Prev APJCP : Asian Pac J Cancer Prev APJCP.

[42] Borka K, Kaliszky P, Szabó E (2007). Claudin expression in pancreatic endocrine tumors as compared with ductal adenocarcinomas. Virchows Arch : an international journal of pathology.

[43] Pyo JS, Kim NY, Cho WJ (2019). Prognostic role of Claudin-1 immunohistochemistry in malignant solid tumors: a meta-analysis. Journal of pathology and translational medicine.

[44] Tsukita S, Tanaka H, Tamura A (2019). The claudins: from tight junctions to biological systems. Trends Biochem Sci.

[45] Katoh M, Katoh M (2024). Claudin 1, 4, 6 and 18 isoform 2 as targets for the treatment of cancer (Review). Int J Mol Med.

[46] Yang KC, Kalloger SE, Aird JJ (2021). Proteotranscriptomic classification and characterization of pancreatic neuroendocrine neoplasms. Cell Rep.

